# High Prevalence of CTX-M-15-Type ESBL-Producing *E. coli* from Migratory Avian Species in Pakistan

**DOI:** 10.3389/fmicb.2017.02476

**Published:** 2017-12-12

**Authors:** Mashkoor Mohsin, Shahbaz Raza, Katharina Schaufler, Nicole Roschanski, Fatima Sarwar, Torsten Semmler, Peter Schierack, Sebastian Guenther

**Affiliations:** ^1^Institute of Microbiology, University of Agriculture, Faisalabad, Pakistan; ^2^Institute of Microbiology and Epizootics, Freie Universität Berlin, Berlin, Germany; ^3^Institute of Animal Hygiene and Environmental Health, Freie Universität Berlin, Berlin, Germany; ^4^NG 1-Microbial Genomics, Robert Koch Institute, Berlin, Germany; ^5^Faculty of Environment and Natural Sciences, Brandenburg University of Technology Cottbus-Senftenberg, Senftenberg, Germany

**Keywords:** antimicrobial resistance, wild birds, ESBL-producing *E. coli*, genomic epidemiology, Pakistan

## Abstract

The increased presence of clinically relevant multidrug resistant bacteria in natural environments is an emerging challenge for global health care. Little is known regarding the occurrence of extended-spectrum beta-lactamase producing *Escherichia coli* (ESBL-*E. coli*) from environmental sentinels in Pakistan. The goal of the current study was to gain insights into the prevalence and phylogenetic relationships of ESBL-*E. coli* recovered from wild birds in Pakistan during winter migration. After initial screening of fecal samples on selective chromogenic agar, ESBL-*E.coli* were analyzed phenotypically using the Vitek-2 automated system. Genotypic characterization was performed using whole genome sequencing (WGS) followed by an in-depth in silico analysis. Of 150 birds screened, 26 (17.3%) were fecal carriers of ESBL-*E. coli*. Of these, 88.4% isolates exhibited multidrug resistance (MDR) phenotypes. Resistance to cefotaxime, ceftazidime, ampicillin, doxycycline, tetracycline and sulfamethoxazole/trimethoprim (CTX-CAZ-AM-DC-TE-SXT) represented the most common pattern of MDR (76.9%). WGS data analysis found *bla*_CTX-M-15_ as the predominant ESBL genotype (92.3%). Other genes encoding resistance to sulfonamides (*sul1/sul2/sul3*), aminoglycosides (*strA, strB, aadA1, aadA2, aadA5, aac(3)-IId-like, aac(3)-IVa-like* and *aph(4)-Ia)*, trimethoprim *(dfrA14* or *dfrA17)*, tetracyclines [*tet(A)/tet(B)*], and fluoroquinolones (*qnr*S1) were detected commonly, often encoded on IncF-type plasmids (76.9%). ESBL-*E. coli* were assigned to 17 different sequence types (STs) of which ST10 and ST7097 (4 isolates each) were the most abundant followed by ST4720, ST93, and ST1139 (2 isolates each). Core-genome phylogeny of the isolates found low numbers (0–29) of single nucleotide polymorphisms (SNPs) in isolates belonged to ST7097 originated from two different locations (Chashma barrage and Rasul barrage). Similar trends were found among isolates belong to ST1139. In addition, WGS-based plasmid typing and S1-digestion found plasmids of the same pMLST type (IncF[F-:A-:B53]) and similar sizes in different bacterial and avian hosts suggesting horizontal gene transfer as another possibility for the spread of ESBL-*E. coli* in avian wildlife in Pakistan.

## Introduction

The intensive use of antimicrobials in human and veterinary medicine has resulted in an emergence of antimicrobial resistance (AMR) in humans, animals and the environment at large (Radhouani et al., 2014; Berendonk et al., 2015). Enterobacteriaceae producing ESBLs have increasingly emerged due to the widespread use of cephalosporins and represent a major challenge in infection control (Pitout and Laupland, 2008). Currently, the most commonly encountered ESBL enzyme is the plasmid-encoded CTX-M-type. In particular, an *E. coli* clone of sequence type 131 (ST131) carrying the CTX-M-15 ESBL has been commonly found in clinical and non-clinical settings (Nicolas-Chanoine et al., 2014).

Previous studies have suggested the environment including water, soil and wildlife as the source for clinically relevant ESBL-*E. coli* (Wright, 2010; Blaak et al., 2015; Guenther et al., 2017), thereby possibly transmitting certain ESBL-*E. coli* clonal lineages or ESBL-plasmids from natural environments to humans, livestock or companion animals. Wild migratory birds have been discussed as sentinels and a potential vectors for the transboundary spread of ESBL- producing bacteria (Raza et al., 2017). Furthermore, wildlife has been considered as reservoir of potentially zoonotic extra-intestinal pathogenic *E. coli* (ExPEC) strains in earlier studies (Ewers et al., 2009; Gordon and Cowling, 2012).

Recently, it has been suggested that certain clonal lineages distinguished by very low number of single nucleotide polymorphisms (SNPs) circulate at the human-animal-environment interfaces which strongly supports the One Health perspective of AMR (Falgenhauer et al., 2016; Schaufler et al., 2016). Pakistan is among the Asian countries that harbor a large number of migratory birds during winter migration along the Indus route coming from Siberia and Central Asia. In this study, we screened wild migratory birds from four different wetland habitats along the Indus migration route in Pakistan to assess the prevalence of ESBL-*E. coli* and to subsequently characterize them in-depth via whole genome sequencing to assess AMR genes, multi locus sequence types (MLST), plasmid replicon types, and virulence-associated genes (VAGs). Additionally, the core genomes of identical STs were analyzed for SNPs.

## Materials and methods

### Sample collection and isolation of ESBL- *E. coli*

**Figure 1:**
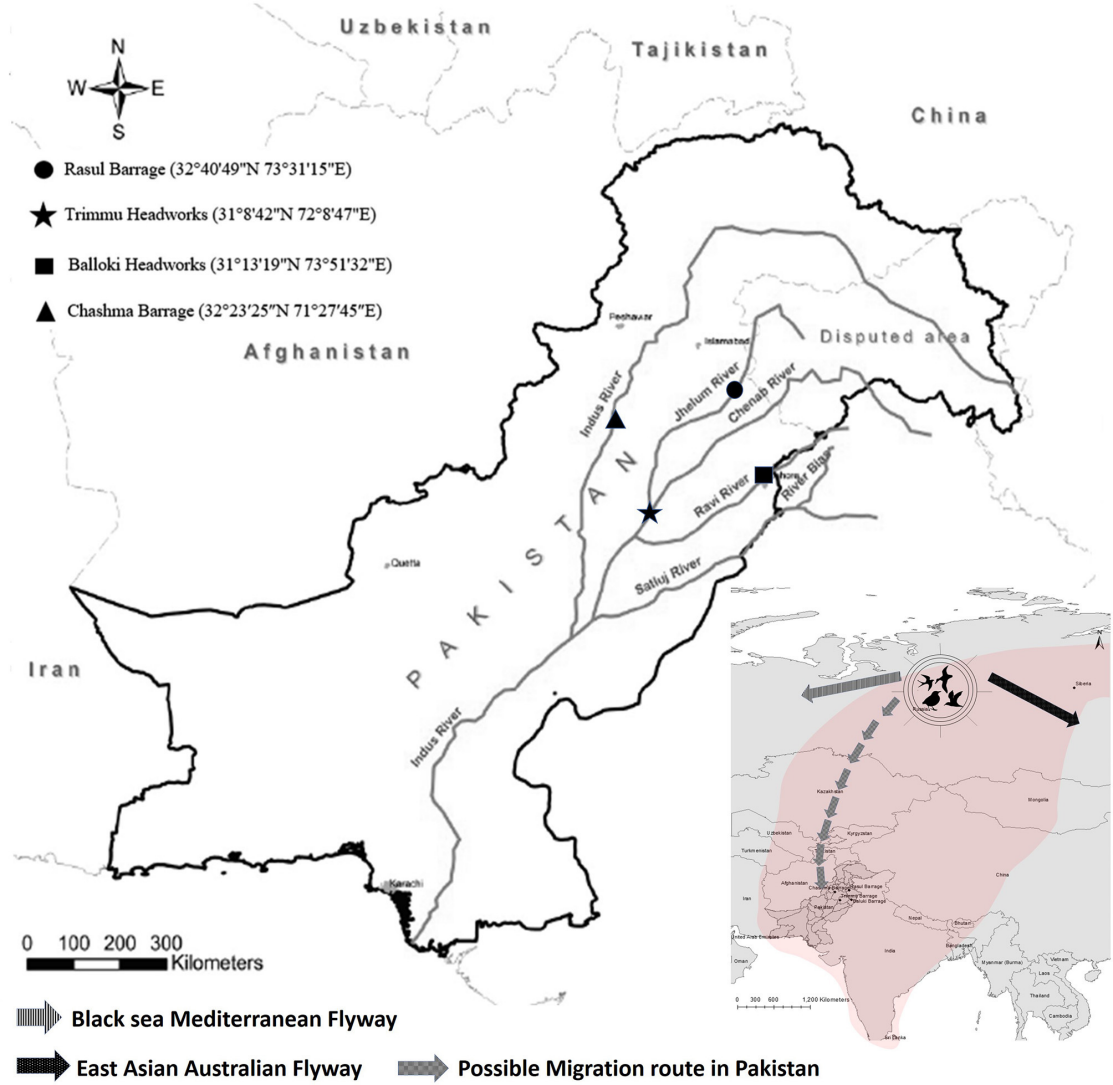
Sampling location and migratory routes of wild birds studied.

In a study conducted between 2013 and 2015, fecal samples of 150 wild migratory birds were collected from four wetland habitats in Pakistan (Figure 1; Raza et al., 2017). These birds included Eurasian coot (*Fulica atra*: *n* = 60), mallard duck (*Anas platyrhynchos*: *n* = 20), common pochard (*Aythya farina*: *n* = 15), red headed pochard (*Netta rufina*: *n* = 10), shoveler duck (*Anas clypeata*: *n* = 15), Eurasian wigeon (*Anas penelope*: *n* = 15) and rosy starling (*Pastor roseus*: *n* = 15). Fecal samples were directly streaked on CHROMagar-ESBL plates (CHROMagar Co., Paris, France) and incubated at 37°C overnight. One putative *E. coli* colony per sample was selected and confirmed by API 20E biochemical strips (bioMérieux, Marcy l'Etoile, France).

### ESBL confirmation and antimicrobial susceptibility testing

Confirmation of the ESBL production was done by double disc synergy test according to the CLSI guidelines (CLSI, 2012) and approved using the Vitek-2 compact system (AST-card GN38, bioMérieux, Germany), which was also used for analyzing additional phenotypic AMRs. Multi-drug resistance (MDR) was defined as resistance to three or more different classes of antimicrobials (Magiorakos et al., 2012).

### Whole genome sequencing

DNA extraction of confirmed ESBL-*E. coli* isolates were performed using MasterPure™ Purification Kit (Epicenter Biotechnologies, WI) according to the manufacturer's instruction. Whole genome sequencing (WGS) and assembly of reads was performed as previously described (Schaufler et al., 2016; Guenther et al., 2017). Briefly, WGS was performed on an Illumina MiSeq (Illumina, San Diego, CA) using an Illumina Nextera XT library with 300 bp paired-end sequencing. Quality control (QC) was performed using the NGS tool kit (70% of bases with a phred score >20). QC report from the assembled genomes has been provided in (Table S1). *De novo* assembly of high-quality filtered reads into contiguous sequences (contigs) and nodes was done using SPAdes. For each *E. coli* analyzed by WGS, a minimum 90-fold coverage was yielded.

### *In silico* analysis

WGS data from multiple bacterial isolates were analyzed simultaneously for their multi-locus sequence types (MLSTs), antibiotic resistance genes, plasmid replicon types and pMLST using the Bacterial Analysis Pipeline Tool at the web service of Center for Genomic Epidemiology (http://www.genomicepidemiology.org/) (Thomsen et al., 2016). In the case of quinolone resistance genes *gyrA* and *parC* detection, the Resistance Gene Identifier (RGI) tool of CARD (Comprehensive Antibiotic Resistance Database) was used (McArthur et al., 2013). Virulence associated genes (VAGs) were detected with an in-house reference sequence collection which maps Illumina reads against chromosomal and plasmid virulence genes found in the Virulence Factor Database for *E. coli* (http://www.mgc.ac.cn/VFs/). In case of strains lacking plasmids, the chromosomal location of the *bla*_CTX−M_ gene was also analyzed with Geneious v. 7.1.2 (Guenther et al., 2017).

For phylogenetic analysis, SNPs between the core genome of isolates were calculated using Harvest suite 1.0 (parsnp) (Treangen et al., 2014) and the number of SNPs in any two isolates were calculated using distance matrix generated in MEGA 7.0 Software (http://www.megasoftware.net/). The phylogenetic tree of the core genomes was visualized using iTOL 3 (http://itol.embl.de/) (Letunic and Bork, 2016).

### S1 digestion

Isolates displaying the pMLST type IncF[F-:A-:B53] were analyzed by S1-nuclease PFGE (Guerra et al., 2004) using the following running conditions: 1–25 s, 17 h, 6 V/cm, 120 V.

## Results

### Prevalence and phenotypic resistance of ESBL-producing *E. coli*

Twenty-six of 150 birds were fecal carriers of ESBL-producing *E. coli* (17.3%), which correspond to six different avian species spread across all sampling areas (Table 1). Of 26 ESBL- producing *E. coli* isolates, 23/26 (88.4%) showed a MDR phenotype. The most common MDR phenotype was cefotaxime, ceftazidime, ampicillin, doxycycline, tetracycline and sulfamethoxazole /trimethoprim (CTX-CAZ-AM-DC-TE-SXT) found in 20/26 (76.9%) isolates (Table 1). In general, trimethoprim/sulfamethoxazole resistance was the most common non-beta-lactam phenotype (92.3%) followed by resistance to tetracycline (84.6%), doxycycline (80.7%), marbofloxacin and enrofloxacin (15.3%). One of these isolates (Pk-13) showed resistance to colistin and has been reported in our previous publication (Mohsin et al., 2016; Table 1). All isolates were susceptible to carbapenems.

**Table 1:** Characteristics of the ESBL producing *E. coli* isolates from wild migratory birds in Pakistan.

**Sample ID**	**Host Species**	**Date of Isolation**	**Sampling location**	**Antibiotic resistances**	**Beta-lactam genes**	**Colistin**	**Aminoglycoside**	**Sulphonamide**	**Quinolone**	**Trimethoprim**	**Tetracycline**	**Phenicol**	**Fosfomycin**	**ST**	**Plasmid replicon types**	**pMLST summary**	**S1-digest plasmid sizes**	**VAGs**
Pk-1	Rosy Starling *(Pastor roseus)*	16/12/2013	Balloki Headworks	CTX, CAZ, AMP, TE, SXT	*blaCTX-M-15, blaTEM-1B*		*strA,strB*	*sul2*	*QnrS1*	*dfrA14-like*	*tet(A)-like*			ST-202	IncFIB	IncF[F-:A-:B53]	n.d.	*astA (East-1), malX, ompA*
Pk-2	Rosy Starling *(Pastor roseus)*	16/12/2013	Balloki Headworks	CTX, CAZ, AMP, C, DC, TE, ENR, MRB, SXT	*blaCTX-M-15*		*aadA1,aadA2*	*sul3*		*dfrA12*	*tet(A)-like*	*cmlA1-like*		ST-224	No replicon		n.d.	*matA (ecpR), astA (East-1), malX, ompA*
Pk-3	Rosy Starling *(Pastor roseus)*	01/01/2014	Trimmu headworks	CTX, CAZ, AMP, DC, TE, SXT	*blaCTX-M-15, blaTEM-1B*		*strA,strB*	*sul2-like*	*QnrS1*	*dfrA14-like*	*tet(A)*			ST-10	IncFIB	IncF[F-:A-:B53]	130kb	*fimC, matA (ecpR), astA (East-1), malX, ompA*
Pk-4	Red-headed pocahard (*Netta rufina*)	16/12/2013	Balloki headworks	CTX, CAZ, AMP, DC, TE, SXT	*blaCTX-M-15, blaTEM-1B*		*strA,strB*	*sul2*	*QnrS1*	*dfrA14-like*	*tet(A)*			ST-10	IncFIB, IncI1	IncF[F-:A-:B53], IncI1[Unknown ST]	110/90kb	*fimC, sitA, astA (East-1), malX, ompA*
Pk-5	Eurasian coot (*Fulica atra*)	11/02/2014	Chashma barrage	CTX, CAZ, AMP, DC, TE, SXT	*blaCTX-M-15, blaTEM-1B*		*strA,strB*	*sul2*	*QnrS1*	*dfrA14-like*	*tet(A)*			ST-10	IncY		n.d.	*fimC, fyuA, irp2, astA (East-1), malX, ompA*
Pk-6	Eurasian coot (*Fulica atra*)	11/02/2014	Chashma barrage	CTX, CAZ, AMP,DC, TE, SXT	*blaCTX-M-1, blaTEM-1C*		*aadA5,strA,strB*	*sul2*		*dfrA17*	*tet(A)*			ST-4720	IncFIC, IncI1, IncFIB, IncFII	IncI1[ST-3], IncF[F18:A-:B1]	n.d.	*bfpm, fimC, metaA (ecpR), tsh, astA (East-1), fyuA, iroN, irp2, iucD, iutA, sitA, sitB, sitC, sitD, cvi, traT, ompA, malX*
Pk-7	Eurasian coot (*Fulica atra*)	11/02/2014	Chashma barrage	CTX, CAZ, AMP, DC, TE, SXT	*blaCTX-M-1, blaTEM-1C*		*aadA5,strA,strB-like*	*sul2*		*dfrA17*	*tet(A)-like*			ST-4720	IncFIC, IncI1, IncFIB, IncFII	IncI1[ST-3], IncF[F18:A-:B1]	n.d.	*bfpm, fimC, metaA (ecpR), tsh, astA (East-1), fyuA, iroN, iucD, iutA, sitA, sitB, sitC, sitD, cvi, traT, ompA, malX*
Pk-8	Eurasian coot (*Fulica atra*)	20/01/2014	Chashma barrage	CTX, CAZ, AMP, DC, TE, SXT	*blaCTX-M-15, blaTEM-1B*		*strA,strB*	*sul2*	*QnrS1*	*dfrA14-like*	*tet(A)*			ST-7097	IncFIB	IncF[F-:A-:B53]	110/90kb	*astA (East-1), sitA, sitB, sitC, sitD, malX, ompA*
Pk-9	Eurasian coot (*Fulica atra*)	20/01/2014	Chashma barrage	CTX, CAZ, AMP, DC, TE, SXT	*blaCTX-M-15*		*strA,strB*	*sul2*	*QnrS1*	*dfrA14-like*	*tet(B)*			ST-1722	No replicon		n.d.	*fimC, matA (ecpR), sfaX, astA (East-1), chuA, malX, ompA*
Pk-10	Red-headed pocahard (*Netta rufina*)	16/12/2013	Balloki headworks	CTX, CAZ, AMP	*blaCTX-M-15*				*QnrS1*					ST-58	No replicon		n.d.	*matA (ecpR), astA (East-1), malX, ompA*
Pk-11	Eurasian coot (*Fulica atra*)	11/02/2014	Chashma barrage	CTX, CAZ, AMP	*blaCTX-M-15, blaTEM-33-like*									ST-361	IncFIC, IncFIB, IncY	IncF[F46^*^:A-:B16]	n.d.	*matA (ecpR), ompA*
Pk-12	Eurasian coot (*Fulica atra*)	11/02/2014	Chashma barrage	CTX, CAZ, AMP, GM, TM, C, DC, TE, SXT	*blaCTX-M-15*		*aac(3)-IVa-like, aph(4)-Ia, strA-like, strB-like*	*sul2*		*dfrA14-like*	*tet(A)*	*catA2-like*	*fosA*	ST-602	IncFIB, IncFIA, IncFIC, IncFII	IncF[F18:A5:B1]	n.d.	*fimC, metaA (ecpR), astA (East-1), iroN, iucD, iutA, sitA, sitB, sitC, sitD, cvi, traT, ompA, malX*
Pk-13	Eurasian coot (*Fulica atra*)	11/02/2014	Chashma barrage	CTX, CAZ, AMP, CO, PO, DC, TE, ENR, MRB, SXT	*blaCTX-M-15, blaTEM-1B*	*mcr-1*	*aadA1,aadA2-like,strA,strB*	*sul2,sul3*		*dfrA14-like*	*tet(B)*	*cmlA1-like*		ST-354	IncFII, IncHI2, IncFIB, IncFIA, IncI2	IncHI2[ST-3], IncF[F36:A6^*^:B1]	n.d.	*fimC, metaA (ecpR), astA (East-1), chuA, iroN, iucD, iutA, sitB, sitC, cvi, ompA, malX*
Pk-14	Eurasian coot (*Fulica atra*)	11/02/2014	Chashma barrage	CTX, CAZ, AMP, DC, TE, SXT	*blaCTX-M-15,blaTEM-1B*		*strA,strB*	*sul2*	*QnrS1*	*dfrA14-like*	*tet(A)*			ST-10	IncY		n.d.	*astA (East-1), fyuA, irp2, malX, ompA*
Pk-15	Mallard duck (*Anas platyrhynchos*)	16/02/2015	Rasul barrage	CTX, CAZ, AMP, DC, TE, SXT	*blaCTX-M-15, blaTEM-1B*		*strA,strB*	*sul2*	*QnrS1*	*dfrA14-like*	*tet(A)*			ST-1139	IncFIB, p0111	IncF[F-:A-:B53]	130/100kb	*astA (East-1), malX, ompA*
Pk-16	Shoveler duck (*Anas clypeata*)	16/12/2013	Balloki headworks	CTX, CAZ, AMP, GM, TM, ENR, MRB, SXT	*blaCTX-M-15, blaTEM-1B*		*aac(3)-IId-like,aadA2,strA-like,strB*	*sul1,sul2*		*dfrA12*				ST-617	ColRNAI		n.d.	*matA (ecpR), astA (East-1), malX, ompA, tia*
Pk-17	Shoveler duck (*Anas clypeata*)	16/12/2013	Balloki headworks	CTX, CAZ, AMP, DC, TE, SXT	*blaCTX-M-15, blaTEM-1B*		*strA,strB*	*sul2*	*QnrS1*	*dfrA14-like*	*tet(A)*			ST-1303	IncFIB	IncF[F-:A-:B53]	130kb	*matA (ecpR), astA (East-1), fyuA, irp2, malX, ompA*
Pk-18	Eurasian wigeon (*Anas penelop*)	01/01/2014	Trimmu headworks	CTX, CAZ, AMP, SXT	*blaCTX-M-15, blaTEM-1B-like*			*sul2*	*QnrS1*	*dfrA1*				ST-2914	IncFII, IncQ1, IncB/O/K/Z	IncF[F55^*^:A-:B-]	n.d.	*matA (ecpR), astA (East-1), chuA, kpsMT_ll, traT,malX, ompA*
Pk-19	Eurasian wigeon (*Anas penelop*)	01/01/2014	Trimmu headworks	CTX, CAZ, AMP, DC, TE, SXT	*blaCTX-M-15, blaTEM-1B*		*strA,strB*	*sul2*	*QnrS1*	*dfrA14-like*	*tet(A)*			ST-3716	IncFIB	IncF[F-:A-:B53]	130kb/ 40kb	*astA (East-1), malX, ompA*
Pk-20	Mallard duck (*Anas platyrhynchos*)	01/01/2014	Trimmu headworks	CTX, CAZ, AMP, DC, TE, ENR, MRB, SXT	*blaCTX-M-15, blaTEM-1B*		*strA,strB*	*sul2*	*QnrS1*	*dfrA14-like*	*tet(A)*			ST-1421	IncFIB	IncF[F-:A-:B53]	130/30kb	*astA (East-1), malX, ompA*
Pk-21	Mallard duck (*Anas platyrhynchos*)	01/03/2014	Chashma barrage	CTX, CAZ, AMP, DC, TE, SXT	*blaCTX-M-15, blaTEM-1B*		*strA,strB*	*sul2*	*QnrS1*	*dfrA14-like*	*tet(A)*			ST-7097	IncFIB	IncF[F-:A-:B53]	130/ 40/30kb	*astA (East-1), sitA, sitB, sitC, sitD, malX, ompA*
Pk-23	Mallard duck (*Anas platyrhynchos*)	01/03/2014	Chashma barrage	CTX, CAZ, AMP, DC, TE, SXT	*blaCTX-M-15, blaTEM-1B*		*strA,strB*	*sul2*	*QnrS1*	*dfrA14-like*	*tet(A),tet(B)*			ST-93	IncFIB	IncF[F-:A-:B53]	60kb	*hek/hra, matA (ecpR), astA (East-1), sitA, sitB, sitC, sitD, kpsMT_ll, malX, ompA*
Pk-24	Mallard duck (*Anas platyrhynchos*)	16/02/2015	Rasul barrage	CTX, CAZ, AMP, DC, TE, SXT	*blaCTX-M-15,blaTEM-1B*		*strA,strB*	*sul2*	*QnrS1*	*dfrA14-like*	*tet(A)*			ST-7097	IncFIB	IncF[F-:A-:B53]	n.d.	*astA (East-1), sitA, sitB, sitC, sitD, malX, ompA*
Pk-26	Eurasian coot (*Fulica atra*)	01/03/2014	Chashma barrage	CTX, CAZ, AMP, DC, TE, SXT	*blaCTX-M-15, blaTEM-1B*		*strA,strB*	*sul2*	*QnrS1*	*dfrA14-like*	*tet(A)*			ST-7097	IncFIB	IncF[F-:A-:B53]	130/100kb	*astA (East-1), sitA, sitB, sitC, sitD, malX, ompA*
Pk-29	Eurasian coot (*Fulica atra*)	01/03/2014	Chashma barrage	CTX, CAZ, AMP, DC, TE, SXT	*blaCTX-M-15, blaTEM-1B*		*strA,strB*	*sul2*	*QnrS1*	*dfrA14-like*	*tet(A)*			ST-1139	IncFIB, p0111	IncF[F-:A-:B53]	130/ 00kb	*astA (East-1), malX, ompA*
Pk-30	Eurasian coot (*Fulica atra*)	01/03/2014	Chashma barrage	CTX, CAZ, AMP, DC, TE, SXT	*blaCTX-M-15, blaTEM-1B*		*strA,strB*	*sul2*	*QnrS1*	*dfrA14-like*	*tet(A),tet(B)*			ST-93	IncFIB	IncF[F-:A-:B53]	60kb	*hek/hra, matA (ecpR), astA (East-1), sitA, sitB, sitC, sitD, kpsMT_ll, malX, ompA*

AMP, ampicillin; C, chloramphenicol; CO, colistin, DC, doxycycline; ENR, enrofloxacin; GM, gentamicin; MRB, marbofloxacin; SXT, PO, polymyxin; sulfamethoxazole/trimethoprim; TE, tetracycline; TM, tobramycin astA (East-1), heat stable cytotoxin associated with enteroaggregative E. coli; malX, phosphotransferase system enzyme II; matA, ecp operon encodes EcpR; ompA, outer membrane protein A; fimC, Type 1 fimbria; sitA, sitB, sitC, sitD, Salmonella iron transport system; bfpm, bundle-forming pilus morphogenesis; fyuA, yersiniabactin receptor; irp2, iron repressible protein; tsh, temperature sensitive hemagglutinin; iroN, siderophore receptor; iucD, aerobactin; iutA, iron uptake transport; cvi, structural genes of colicin V operon; traT, transfer protein; kpsMTT_II, group II capsule antigen; hek/hrA, heat resistant hemagglutinin; chuA, E. coli haem utilization; tia, toxigenic invasion locus; sfaX, fimbriae

### Antibiotic resistance and virulence genes

WGS revealed that all of 26 ESBL-*E. coli* isolates harbored the *bla*_CTX−M_ gene with *bla*_CTX−M_-15 as the most dominant 24/26 (92.3%) genotype (Table 1). Of these, 19 isolates also harbored *bla*_TEM−1B_ whereas two isolates carried *bla*_CTX−M−1_ together with *bla* TEM-1C. Among non-beta-lactam resistance, genes conferring resistance to sulfonamide and trimethoprim were predominant 24/26 (92.3%) followed by aminoglycosides 23/26 (88.4%), tetracycline 22/26 (84.6%) and quinolones 19/26 (73%). We found that most of the isolates carried the *sul2* gene, alone or in combination with *sul1* or *sul3* for sulfonamide resistance. A total of 7 different genes encoding resistance for aminoglycoside were detected. Of these, most common were *strA* and *strB*, alone or in combination with *aadA1, aadA2, aadA5, aac(3)-IId-like, aac(3)-IVa-like*, and *aph(4)-Ia*. Overall, genotypic data strongly correlated with phenotypic resistance data. Virulence gene analysis exhibited an overall low number of VAGs in wild birds studied. ExPEC were defined as suggested previously which is mainly based on the presence of at least two VAGs including P fimbrial genes *papA* and *papC*, S frimbriae genes *sfa/foc*, afimbrial adhesion genes *afa/dra*, group 2 polysaccharide capsule gene *kpsMTII* and iron acquisition gene *iutA* (Nowak et al., 2017). According to this definition, none of the isolates is regarded as ExPEC (Table 1). All isolates contained *E. coli* outer membrane protein A gene (*ompA*). Other common genes were *malX, astA* and *iha* coding phosphotransferase system enzyme II, enteroaggregative heat-stable toxin EAST1 and iron-regulated-gene-homologue adhesion, respectively.

### MLST, plasmid replicon types and plasmid profile analysis

In this study, 17 different STs were observed among the 26 sequenced ESBL-*E. coli*. Among the known STs, the most common ones were ST10 and ST7097 (each n = 4) followed by ST4720, ST93, and ST1139 (2 isolates each) whereas one isolate each of ST1421, ST354, ST224, ST1303, ST2914, ST202, ST602, ST58, ST617, ST361, ST3716, and ST1722 were found (Table 1). In silico plasmid replicon typing revealed the IncF-type plasmid as the most common (20/26; 76.9%). The other replicon types detected in this study included IncY, IncI1, IncI2, IncHI2, IncQ1, IncB/O/K/Z. Out of 20 isolates with IncF replicon type, 19 belonged to IncFIB class followed by IncFII (*n* = 5), IncFIC (*n* = 4) and IncFIA (*n* = 2). pMLST of the IncF plasmids revealed the presence of one common plasmid type F-:A-:B53 (*n* = 14). Analysis of the plasmid size with S1 digestion showed a 130 kb plasmid in most of the isolates (Table 1). In contrast, no replicons were detected in the Pk-2, Pk-9 and Pk-10 but those isolates harbored *bla*_CTX−M_-15 encoded on large contigs whose annotation pointed toward a chromosomal integration of the resistance gene.

### Whole genome phylogeny

**Figure 2:**
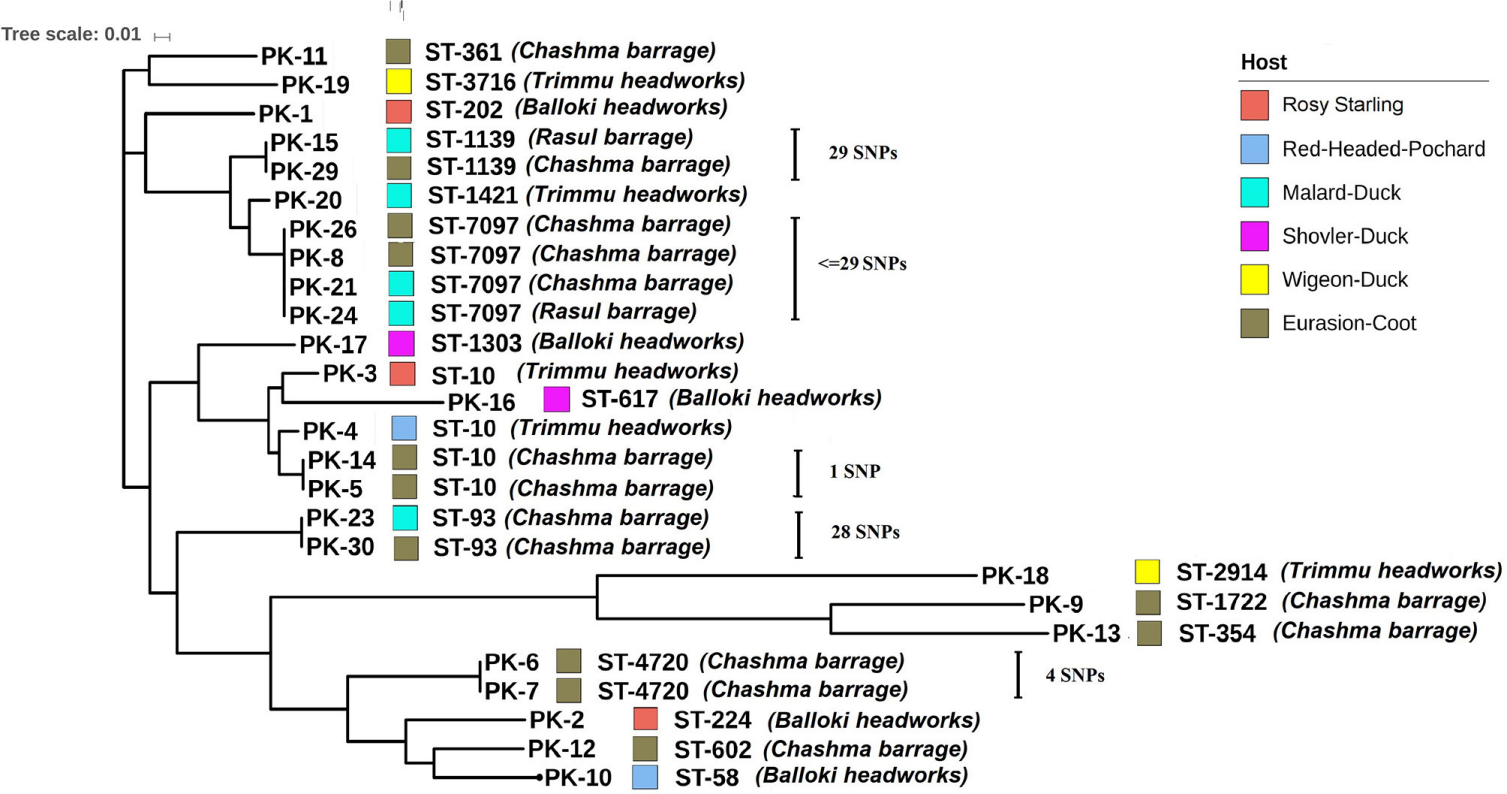
Whole Genome phylogeny based upon core genomes of 26 avian ESBL-*E. coli*. The tree was produced using Harvest Suite and drawn by MEGA5 software.

Core-genome based phylogenetic analysis of 26 isolates grouped *E. coli* into four clusters. Most of the sequenced isolates clustered together in accordance with their ST (Figure 2). Core genome alignment showed very few SNPs ranging from 0 to 29 among isolates Pk-8, Pk-21, Pk-24, and Pk-26 (Figure 2 and Table S2). All of these strains belonged to ST7097 and originated from two different hosts (Eurasian coot and mallard duck) and sampling locations (Chashma barrage and Rasul barrage). Likewise, only 29 SNPs were present between Pk-15 and Pk-29 isolates although recovered from different hosts (Eurasian coot and mallard duck) and locations (Chashma barrage and Rasul barrage). More strikingly, only one SNP was found between Eurasian coot isolates Pk-5 and Pk-14 originated from Chashma barrage. Fewer than 28 SNPs were observed between Pk-23 and Pk-30 (isolated from mallard duck and Eurasian coot from Chashma barrage). Two *bla*_CTX−M−1_-producing *E. coli* Pk-6 and Pk-7 were marked by only four SNPs and were recovered from a similar geographic location and host (Figure 2). Numbers of SNPs for the individual isolates are displayed in Table S2.

## Discussion

Wild migratory birds have been suggested as a reservoir of ESBL-producing *E. coli* in a number of studies worldwide (Guenther et al., 2011, 2012; Bonnedahl et al., 2015; Atterby et al., 2016). More recently, we reported the occurrence of *bla*-_CTX−M−15_ producing *Klebsiella pneumoniae* (Raza et al., 2017) in wild migratory bird populations in Pakistan. We therefore also screened for ESBL-producing *E. coli* and their clonal relatedness using WGS, as there is lack of knowledge regarding genetic diversity of ESBL-*E. coli* isolates from environmental niches in Asia. *E. coli* is an excellent indicator species to study the spread of AMR through fecal pollution of water and waterfowl can be considered as sentinel of AMR in the environment (Guenther et al., 2011). The present study indicates high carriage rates of ESBL-producing *E. coli* (17%) in migratory birds along the Indus migration route in Pakistan. This high prevalence mirrors those reported in migratory gulls from Bangladesh (17.3%) (Hasan et al., 2014) and is comparable to another study from Bangladesh which reported 30% ESBL-*E. coli* from wild ducks (Hasan et al., 2012). This is underlining the important role of waterfowl as carrier of ESBL-producing *E. coli* in Asia and also adding the important Indus avian migration route to the environments influenced by human healthcare practices.

WGS showed *bla*_CTX−M−15_ was the predominant ESBL genotype in this study. This is in agreement with some previous findings from wild birds in Bangladesh (Hasan et al., 2014), Germany (Guenther et al., 2010) and North America (Poirel et al., 2012). CTX-M-15 has now a worldwide distribution and although it is commonly associated with human and pet ESBL-isolates, it is also very common in avian wildlife (Wang et al., 2017).

In fact, summing up the current literature it becomes obvious that the emergence of ESBL-producing *E. coli* in wildlife is associated with the success of the *bla*_CTX−M_ family in hospitals (Guenther et al., 2011). The reason why *bla*_CTX−M_ producing *E. coli* are also very successful in the environment remain unclear but recent studies suggest that plasmids carrying those genes confer more advantages than mere resistance to the bacterial host strains (Schaufler et al., 2016). A previous study also indicated high rates of *bla*_CTX−M−15_ from human clinical isolates in Pakistan (Habeeb et al., 2014), however as we did not include human isolates in this study their relatedness remains to be clarified in the future.

Besides their spread via plasmids, very recently the new trend of chromosomal integration of ESBL-encoding genes has been demonstrated in clinical *E. coli* isolates of ST38, ST410, ST131 and ST648 (Hirai et al., 2013; Rodríguez et al., 2014; Falgenhauer et al., 2016) and also in non-clinical ST38 isolates from wild birds (Guenther et al., 2017). Similarly, we detected the chromosomal insertion of *bla*_CTX−M−15_ genes among *E. coli* of different STs (ST224, ST1722 and ST58), which have been found as plasmid carrying ESBL-producers in clinical and non-clinical samples, worldwide (Zurfluh et al., 2013; Leangapichart et al., 2016). This scenario has also been recently shown for *E. coli* strains of ST38 from Mongolian wild birds, which were very closely related to a clinical outbreak strain from the UK (Guenther et al., 2017).

As mentioned above, wildlife has been reported to carry ExPEC strains, we therefore also screened for the occurrence of VAGs to gain information on pathotype. However, we detected no ExPEC strain in our isolates. Most of the strains harbored only a few VAGs and are likely commensal strains. However, all the *E. coli* carried serum resistance *ompA* gene (Table 1). We also found high frequency of *astA* and *iha* genes. These are only putative virulence genes and their exact involvement in the pathogenesis is not well understood, although they have been frequently reported in enteroaggregative *E. coli* and avian pathogenic *E. coli* (Nowak et al., 2017).

We found a large diversity of sequence types within the avian isolates including typical ESBL-associated sequence types like ST10, ST224, ST617 (Guenther et al., 2011; Sherchan et al., 2015), and ST354 (Zhang et al., 2016). However, globally distributed high risk clones like ST131, ST410, and ST648 were not found in this study. Earlier studies from human clinical *E. coli* isolates from Pakistan reported those sequence types including ST131 and ST648 (Mushtaq et al., 2011; Pesesky et al., 2015), indicating that different clonal population of *E. coli* might be present in wild birds and the human population in Pakistan but this finding can also be due to the low number of birds sampled.

Interestingly we found identical STs in isolates originating from different avian host species and geographic locations (Figure 2). Core genome phylogenetic analysis of those isolates showed that within identical STs only a small number of SNPs ranged from 1 to 29 were found. This suggests a recent interspecies transmission and long-distance dissemination of certain clonal ESBL-lineages by wild birds as it has been reported earlier (Guenther et al., 2017). The origins of most of these birds are remote areas in Siberia and Central Asia and exposure to antimicrobials is less likely in these areas. The high rates of MDR isolates detected from the wild migratory bird are of concern and could be due to anthropogenic activities from the surrounding environment. In addition to the clonal spread of certain STs our data showed the common occurrence of a plasmid replicon type (IncFIB, F-:A-:B53) linked to a 130 kb plasmid. This plasmid was found in all four wetlands tested and in five of the seven different avian species. Together with the large number of minor STs points toward the spread of a *bla*_CTX−M_ resistance plasmid of the pMLST type F-:A-:B53 among a naive *E. coli* population in the avian hosts.

The transmission dynamics of ESBL-producing *E. coli* in a natural environment are complex. Wild birds have been suggested as sentinels for the spread and transmission of multi-resistant strains in the environment. It is widely believed that the spread of ESBL-*E. coli* is driven both by plasmid transfer in commensal and pathogenic strains as well as by the clonal spread of certain lineages in local areas. In this study we were able to detect both main mechanisms in wild migratory birds in Pakistan underlining the suitability of avian sentinels. In addition our data highlights the potential for regional and intercontinental transmission of ESBL-producing *E. coli* clones and resistance plasmids via migratory birds.

## Author contributions

MM, SG: conceived and designed the experiments; MM, SR, and FS: collected the data and samples; MM, KS, NR, FS, and PS: performed laboratory analysis; SG, MM, SR, and TS: analyzed the data; TS and SG: performed WGS; MM and SG: wrote the article. All authors have read and approved the final draft of the manuscript.

### Conflict of interest statement

The authors declare that the research was conducted in the absence of any commercial or financial relationships that could be construed as a potential conflict of interest.
